# Modified Low-Dose Triiodo-L-thyronine Therapy Safely Improves Function Following Myocardial Ischemia-Reperfusion Injury

**DOI:** 10.3389/fphys.2017.00225

**Published:** 2017-04-12

**Authors:** Viswanathan Rajagopalan, Youhua Zhang, Christine Pol, Clifford Costello, Samantha Seitter, Ann Lehto, Olga V. Savinova, Yue-feng Chen, A. Martin Gerdes

**Affiliations:** ^1^Department of Basic Sciences, New York Institute of Technology-College of Osteopathic MedicineJonesboro, AR, USA; ^2^Department of Biomedical Sciences, New York Institute of Technology-College of Osteopathic MedicineOld Westbury, NY, USA

**Keywords:** cardiac physiology, ischemia-reperfusion injury, thyroid hormones, heart function, therapeutic protocol

## Abstract

**Background:** We have shown that thyroid hormones (THs) are cardioprotective and can be potentially used as safe therapeutic agents for diabetic cardiomyopathy and permanent infarction. However, no reliable, clinically translatable protocol exists for TH treatment of myocardial ischemia-reperfusion (IR) injury. We hypothesized that modified low-dose triiodo-L-thyronine (T3) therapy would confer safe therapeutic benefits against IR injury.

**Methods:** Adult female rats underwent left coronary artery ligation for 60 min or sham surgeries. At 2 months following surgery and T3 treatment (described below), the rats were subjected to functional, morphological, and molecular examination.

**Results:** Following surgery, the rats were treated with T3 (8 μg/kg/day) or vehicle in drinking water *ad libitum* following IR for 2 months. Oral T3 significantly improved left ventricular (LV) contractility, relaxation, and relaxation time constant, and decreased beta-myosin heavy chain gene expression. As it takes rats ~6 h post-surgery to begin drinking water, we then investigated whether modified T3 dosing initiated immediately upon reperfusion confers additional improvement. We injected an intraperitoneal bolus of T3 (12 μg/kg) upon reperfusion, along with low-dose oral T3 (4.5 μg/kg/day) in drinking water for 2 months. Continuous T3 therapy (bolus + low-dose oral) enhanced LV contractility compared with oral T3 alone. Relaxation parameters were also improved compared to vehicle. Importantly, these were accomplished without significant increases in hypertrophy, serum free T3 levels, or blood pressure.

**Conclusions:** This is the first study to provide a safe cardiac therapeutic window and optimized, clinically translatable treatment-monitoring protocol for myocardial IR using commercially available and inexpensive T3. Low-dose oral T3 therapy supplemented with bolus treatment initiated upon reperfusion is safer and more efficacious.

## Introduction

Despite advancements, cardiovascular diseases remain the No. 1 disease burden worldwide (Dzau, 2016). In the United States, coronary artery diseases contribute to almost half of the cardiovascular deaths and myocardial ischemia-reperfusion (IR) injury remains a major challenge. Although IR is less injurious than permanent occlusion of the culprit vessel(s), IR also results in significant oxidative stress and functional compromise. The challenge for researchers is to identify interventions that maximize recovery of reperfused myocardium. Thyroid hormone (TH)-mediated cardioprotection has been demonstrated using various models of cardiovascular disorders (Rajagopalan and Gerdes, 2014; Gerdes and Ojamaa, 2016). Both thyroid hormones (THs), Thyroxine (T4) and triiodo-L-thyronine (T3) are secreted systemically by the thyroid gland and perform numerous physiological functions on heart and other systems. Free T3 is the active form of T3 and is a clinically important biomarker. Thyroid-stimulating hormones (TSHs) stimulate TH release and regulate their serum levels. Hsu et al. (1995) has previously shown that T3 supplementation after IR in a pig model significantly enhanced acute post-ischemic left ventricular (LV) functional recovery. However, these results have not been followed long term or translated into patient care. The apparent perception that THs may be risky for clinical therapy is largely based on problematic TH analog studies (Stamler, 1977; Young et al., 1984; Goldman et al., 2009) where the analog was contaminated with active L-thyroxine or excessive dosing likely occurred. In this regard, we request the readers to refer to detailed published reviews by our group and others (Pingitore et al., 2010; Gerdes, 2015; Gerdes and Ojamaa, 2016; Jabbar et al., 2017).

To overcome these challenges, robust translatable protocols that are both safe and efficacious are needed. Recent animal data from our lab and others' suggests that fear of TH overtreatment may actually be causing harm by withholding treatment from many patients who will likely benefit by TH replenishment (Weltman et al., 2014, 2015; Gerdes and Ojamaa, 2016). Although T4 is used for the treatment of patients with primary hypothyroidism, T3, T4, or combination treatment have been proposed for the treatment of hypothyroidism secondary to cardiovascular disorders (Gerdes and Iervasi, 2010; Suh and Kim, 2015). Though the issue is far from being resolved, T3 may be preferred since the conversion mechanisms of T4 into T3 are known to be impaired in non-thyroidal illness syndrome (Pingitore et al., 2012). T3 largely by-passes this potential problem of bioavailability by direct application of the active form of TH (Weltman et al., 2013). We have shown that short-term treatment of T3 protected cardiomyocytes against ischemia-induced apoptosis (Chen et al., 2008). In addition, short-term T3 treatment in patients with ischemic and non-ischemic HF was well-tolerated and resulted in improved cardiac function and attenuated neurohormonal activation (Hamilton et al., 1998; Pingitore et al., 2008). However, long-term protection is unclear in humans.

Recently, we demonstrated that oral T3 treatment for 2 months following myocardial infarction (MI) improved cardiac function and rhythm without significant cardiac adverse effects (Rajagopalan et al., 2016). Additional information is needed regarding therapeutic T3 treatment of IR. We hypothesized that similar protection will be conferred following myocardial IR. This is the first study to demonstrate that a modified low-dose oral T3 therapy in IR is safer for the heart and more efficacious in not only a dose-dependent, but also, a time-dependent manner.

## Materials and methods

### Ethical approval

This study was approved by the Institutional Animal Care and Use Committee of the New York Institute of Technology College of Osteopathic Medicine (NYIT-COM) and is in compliance with the “Guide for the Care and Use of Laboratory Animals” (National Institutes of Health Publication No. 85–23, Revised 1996). Based on ARRIVE guidelines (NC3Rs), adult female Sprague-Dawley rats aged 11 weeks old were purchased from Harlan (Indianapolis, IN, USA), acclimatized and housed up to 3 per cage. They were kept on a 12-h light–dark cycle with standard rat chow and water provided *ad libitum*. Permanent ligation MI procedure was performed by the same surgeon as described before (Chen et al., 2013; Rajagopalan et al., 2016).

### Ischemia-reperfusion injury

Rats were randomly assigned to a sham-operated group or an IR group. The operating surface was prepared by disinfecting the area with 70% ethanol. All surgical instruments were sterilized with a hot bead sterilizer before surgery and in between individual rat surgeries. These instruments include: surgical scissors, forceps, needle holders, and a chest retractor. Heating pad using a circulating water T/Pump (Gaymar Industries Inc., Orchard Park, NY) was used to maintain the body temperature of the animal at 37 ± 1°C. An electric shaver was used to shave the fur from the neck and chest areas. Shaved areas were scrubbed and disinfected with betadine solution followed by wiping the area with 70% alcohol. Anesthetized animal was placed in a supine position on the heating pad. Endotracheal intubation was performed and mechanical ventilation was achieved by connecting the endotracheal tube to a Kent Scientific ventilator cycling at 70 breaths per minute and a tidal volume of 1.0 ml per 100 gram body weight. IR was induced by 60 min ligation of the left anterior descending coronary artery. At the end of the ischemic period, the knot around the blood vessel was loosened and unrestrained reperfusion was allowed. The silk suture was left *in situ*, the chest retractor was removed and the ribs were drawn together using a 5-0 silk suture with a continuous suture pattern. Sham-operated animals underwent the same procedure except that the suture was tied loosely around the coronary artery. Tidal volume of the ventilator was temporarily increased to expand the lungs and expel air from the chest. The skin was closed using 5-0 silk in a continuous suture pattern.

Following extubation, intramuscular Buprenorphine analgesia was administered. IR rats were randomly assigned to an IR+V (vehicle) group and an IR+T3 group. T3 (0.08 μg/ml) was dissolved in ethanol/glycerol and added to drinking water for a total duration of 2 months (mo). This provided a dose of 8 μg/kg/d based on daily water consumption (Rajagopalan et al., 2016) and serum TH feedback inhibition response from previous studies (Weltman et al., 2014). Vehicle was used in rats not treated with T3 using the aforementioned diluents. In a subsequent cohort of IR rats, we further repeated studies of oral T3 (at 0.045 μg/ml or 4.5 μg/kg/d) along with an intraperitoneal (i.p.) bolus of T3 (12 μg/kg) upon reperfusion. Based on results of the first experiment, the oral T3 dose was reduced for optimization.

At 2 mo post-IR, blinded, standard isoflurane-anesthetized (3% induction, 1.5% maintenance) cardiac catheterization studies were followed by body weight measurements and terminal experiments (surgical plane isoflurane anesthesia followed by diastolic cardiac arrest). This included blood collection for serum TH levels and tissue collection for histology and molecular studies.

### Echocardiography

Two-dimensional echocardiograms (GE Vivid 7 Dimension, Horten, Norway; M12L transducer) were obtained from LV short-axes to visualize the extent of infarction in the immediate post-op period (Chen et al., 2013). Animals that did not develop infarcts between 30 and 50% (Pfeffer et al., 1985) and those that did not survive for more than 48 h (immediate post-op period) were excluded (Rajagopalan et al., 2016). Subsequently, at 2-mo post-surgery, structural assessments were performed.

### Serum thyroid hormone levels

LV blood samples were collected, separated into serum by centrifugation, and stored at −80°C until assayed. Serum TH levels were measured using commercial enzyme-linked immunosorbent assay (ELISA) kits (TSH: ALPCO, Salem, NH, USA; T3, FT3, T4, FT4: Monobind Inc., Lake Forest, CA, USA) according to manufacturer specifications and prior studies (Weltman et al., 2014; Rajagopalan et al., 2016).

### Hemodynamics

LV hemodynamic data were obtained under anesthesia by right carotid arterial catheterization using a 1.9F SciSense pressure-volume catheter (Transonic Scisense Inc., London, Ontario). The catheter tip was advanced through the aorta into the LV to study arterial and LV pressures, change in pressures over time (dP/dt) and other hemodynamic parameters. The data were acquired and analyzed by LabScribe (iWorx Systems, Inc., Dover, NH).

### Electrophysiology studies

Electrophysiological studies were performed using techniques successfully employed for rat myocardial ischemic and thyroid models (Zhang et al., 2013, 2014; Rajagopalan et al., 2016). Under anesthesia, a 1.6F octopolar Millar electrophysiology catheter (EPR-802, Millar Instruments, Inc., Houston, Texas) was inserted through right jugular vein and advanced into the right atrium to record atrial electrograms. Standard surface ECG lead II and 3 right atrial electrocardiograms were displayed and recorded using a PowerLab data acquisition system (ADInstruments, Colorado Springs, CO). Burst pacing containing 200 impulses at 50 Hz was used to induce atrial tachyarrhythmias (ATA). ATA were defined as rapid atrial activations with varying electrogram morphology lasting >0.5 s. The mean ATA duration based on 5 such tests was used to reflect the ATA substrate in each animal.

### Histology

Hearts were arrested in diastole by injection of 0.2 M potassium chloride via the LV apex followed by rapid transfer to ice-cold phosphate buffered saline. Subsequently, aortas were cannulated with an 18G gavage needle to allow coronary perfusion with ice-cold phosphate buffered saline containing 0.2% 2,3-butanedione monoxime. Fat and aorta were trimmed and discarded, hearts blotted, and chamber weights quickly documented. The transverse mid-slice of the LV with septum was dissected. Paraformaldehyde-fixed 5 μm thick LV tissue sections were stained with Masson's Trichrome stain. Images were acquired using Olympus BX53 microscopes and transmural infarct characteristics were analyzed using Image-Pro plus (Media Cybernetics, Bethesda, MD) or Image J (National Institutes of Health, Bethesda, MD) programs.

### Real-time quantitative PCR

RNA isolation and real time PCR was performed following similar protocols as before (Rajagopalan et al., 2016). Briefly, RNA was isolated using TRIzol reagent followed by RNA purification using PureLink RNA mini kit and DNaseI kit (Invitrogen, Carlsbad, CA). RNA quantity and quality were determined and validated using NanoDrop 1000 (Thermo Scientific, Wilmington, DE). Equal amounts of RNA from each sample were converted to cDNA using RT^2^ First Strand Kit (Qiagen Inc., Valencia, CA). Gene expression was evaluated using SYBR green/ROX detection (Supplementary Table) and Applied Biosystems StepOnePlus (Life Technologies Corporation, Carlsbad, CA). GAPDH (Glyceraldehyde 3-phosphate dehydrogenase) and beta-actin or *Ppia* (cyclophilin A) were used as housekeeping control genes and expression data analyzed using 2^−ΔΔCT^ method.

### Statistical analysis

All data are expressed as means ± standard error, unless otherwise noted. Data analysis was performed with GraphPad Prism for Windows (GraphPad, San Diego, CA) and groups were compared using student's *t*-test or one-way analysis of variance (ANOVA) followed by *post-hoc* tests. Fisher exact test was used to compare the incidence of atrial tachyarrhythmias (ATA; Zhang et al., 2013). A non-parametric Kruskal–Wallis test followed by Dunn's multiple comparison test was used to compare ATA duration in the oral treatment groups. A value of *P* < 0.05 was considered statistically significant.

## Results

### Morphometric changes following oral T3 treatment

Although starting body weights were lower in the IR groups [IR+V (O) and IR+T3 (O)] compared to Sham+V (O), all the groups gained weights proportionately over time (Table 1). Two months oral T3 treatment did not affect body weight, as there was no difference between IR+V (O) and IR+T3 (O) groups. IR tended to increase heart weight and significantly increased LV weight (LV+septal weight) and LV weight to body weight ratio compared to sham values. Oral T3 at 8 μg/kg/d following IR further increased these parameters. Histologically, there was no statistically significant difference in viable areas within infarct segments between both IR groups (IR+V: 5.85 ± 0.89 mm^2^; IR+T3: 5.27 ± 0.68 mm^2^; Supplementary Figure 1).

**Table 1:** **Cardiac remodeling in 2 mo IR rats after oral (only) T3**.

	**Sham+V (O)**	**IR+V (O)**	**IR+T3 (O)**
Body weight, peri-op (g)	232 ± 9	216 ± 7^*^	223 ± 16
Body weight, post-op (g)	273 ± 12	259 ± 12	258 ± 15
Heart weight (mg)	938 ± 108	997 ± 82	1,171 ± 212^*^^§^
Heart weight/BW (mg/g)	3.5 ± 0.5	3.8 ± 0.3	4.5 ± 0.6^***^^§§^
LV (+ septum) weight (mg)	654 ± 73	712 ± 49^*^	790 ± 49^***^^§§^
LV (+ septum) weight/BW (mg/g)	2.4 ± 0.3	2.7 ± 0.2^**^	3.1 ± 0.1^***^^§§^

Values are means ± SD; BW, body weight; LV, Left ventricular; RV, right ventricular; O, Oral; V, Vehicle; IR, Ischemia-reperfusion; T3, Triiodo-L-thyronine;

* *p < 0.05*,

** *p < 0.01*,

*** p < 0.001 vs. Sham+V;

§ *p < 0.05*,

§§ *p < 0.01 vs. IR+V; n = 7 (Sham+V), n = 11 (IR+V), n = 9 (IR+T3)*.

### Serum thyroid hormone levels following oral T3

IR did not significantly alter serum thyroid profiles, except increasing free T4 levels (Table 2). T3 treatment at 8 μg/kg/d increased serum free T3 levels. Correspondingly, as expected, T3 resulted in feedback inhibition of both T4 and TSH levels. In addition, oral T3 treatment restored the free T4 levels following IR.

**Table 2:** **Serum thyroid hormone levels in 2 mo IR rats after oral (only) T3**.

	**Sham+V (O)**	**IR+V (O)**	**IR+T3 (O)**
Total T3 (ng/ml)	1.1 ± 0.05	1.1 ± 0.2	1.5 ± 0.65
Free T3 (pg/ml)	2.6 ± 1	3.1 ± 0.5	4.0 ± 0.9^**^^§^
Total T4 (ng/ml)	49 ± 6.8	45 ± 12	29 ± 11^**^^§§^
Free T4 (pg/ml)	8.9 ± 4.6	16 ± 3.4^**^	6.5 ± 4.8^^§§§^
TSH (ng/ml)	1.7 ± 0.5	1.8 ± 0.8	0.4 ± 0.3^**^^§§§^

Values are means ± SD; O, Oral; V, Vehicle; IR, Ischemia-reperfusion; T3, Triiodo-L-thyronine; T4, Thyroxine; TSH, Thyroid stimulating hormone;

** p < 0.01, vs. Sham+V;

§ *p < 0.05*,

§§ *p < 0.01*,

§§§ *p < 0.001 vs. IR+V; n = 5–7 (Sham+V), n = 11 (IR+V); n = 9 (IR+T3)*.

### Improvement in cardiac remodeling and function with oral T3

**Figure 1:**
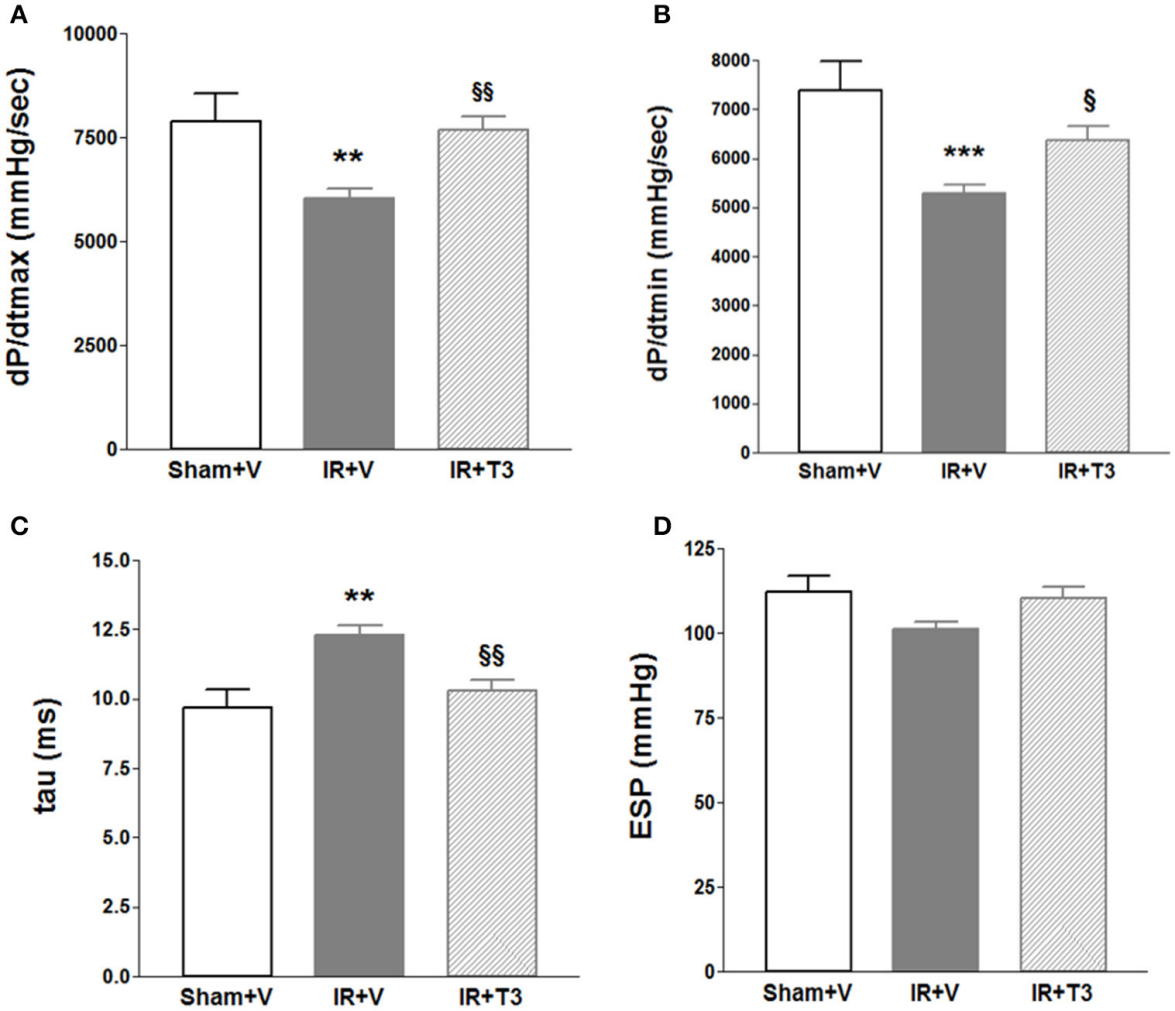
**Hemodynamic changes after oral (only) T3 treatment**. Oral T3 treatment following IR significantly enhanced **(A)** dP/dt_max_, maximal rate of pressure development, **(B)** dP/dt_min_, maximal rate of pressure decline and **(C)** tau, τ, LV relaxation time constant. **(D)** ESP, LV End-systolic pressure is unaffected; V, Vehicle; *n* = 7 (Sham+V), *n* = 11 (IR+V); *n* = 9 (IR+T3); ^**^p < 0.01, ^***^p < 0.001 vs. Sham+V; ^§^*p* < 0.05, ^§§^*p* < 0.01 vs. IR+V.

Heart rate (HR) showed a mild increase following T3 treatment with 8 μg/kg/d by echocardiography (Table 3). LV posterior wall dimension following IR was significantly improved by oral T3 treatment. While IR significantly impaired LV systolic anterior wall thickness, internal dimensions and function, T3 did not significantly affect these parameters. Cardiac catheterization studies (Figures 1A–D) showed that IR resulted in impaired contractility, as indicated by a reduction in maximal rate of LV pressure development (dP/dt_max_; Figure 1A; Supplementary Figure 2). Oral T3 treatment restored this contractility index. Similarly in IR, diastolic function was impaired, reflected by a decrease in maximal rate of pressure decline (dP/dt_min_; Figure 1B) and an increase in LV relaxation time constant, tau (Figure 1C). Both these parameters were significantly improved following oral T3.

**Table 3:** **Echocardiographic parameters in 2 mo IR rats after oral (only) T3**.

	**Sham+V (O)**	**IR+V (O)**	**IR+T3 (O)**
Heart rate (bpm)	343 ± 13	344 ± 33	378 ± 43
LVPWs (mm)	2.7 ± 0.4	2.2 ± 0.6^*^	2.6 ± 0.3^§^
LVPWd (mm)	1.8 ± 0.3	1.7 ± 0.4	2.0 ± 0.2^†^
LVIDs (mm)	3.8 ± 0.7	6.4 ± 1.2^***^	6.7 ± 1.6^***^
LVIDd (mm)	7.1 ± 0.5	8.6 ± 0.9^**^	8.9 ± 1.1^**^
LVAWd (mm)	1.7 ± 0.3	1.3 ± 0.3	1.4 ± 0.5
LVAWs (mm)	2.4 ± 0.6	1.5 ± 0.3^**^	1.6 ± 0.6^**^
FS (%)	46.7 ± 6.8	25.8 ± 6.5^***^	25.8 ± 9.4^***^
EF (%)	82.4 ± 6.3	55.5 ± 10.8^***^	54.6 ± 15.8^***^

Values are means ± SD; LVPW, left ventricular posterior wall; LVAW, left ventricular anterior wall; LVID, left ventricular internal diameter; FS, Fractional shortening; EF, Ejection fraction; s, systole; d, diastole; O, Oral; V, Vehicle; IR, Ischemia-reperfusion; T3, Triiodo-L-thyronine;

* *p < 0.05*,

** *p < 0.01*,

#x0002A;**: p < 0.001 vs. Sham+V;

§ p < 0.05, vs. IR+V;

† *p < 0.05 vs. IR+V (t-test); n = 7 (Sham+V), n = 11 (IR+V); n = 9 (IR+T3)*.

Investigating electrophysiological characteristics of IR hearts, we found significant atrial arrhythmia in only one out of 11 vehicle-treated IR rats. Similar to the sham group, oral T3 treatment resulted in no arrhythmias.

### Expression of representative genes following oral T3

**Figure 2:**
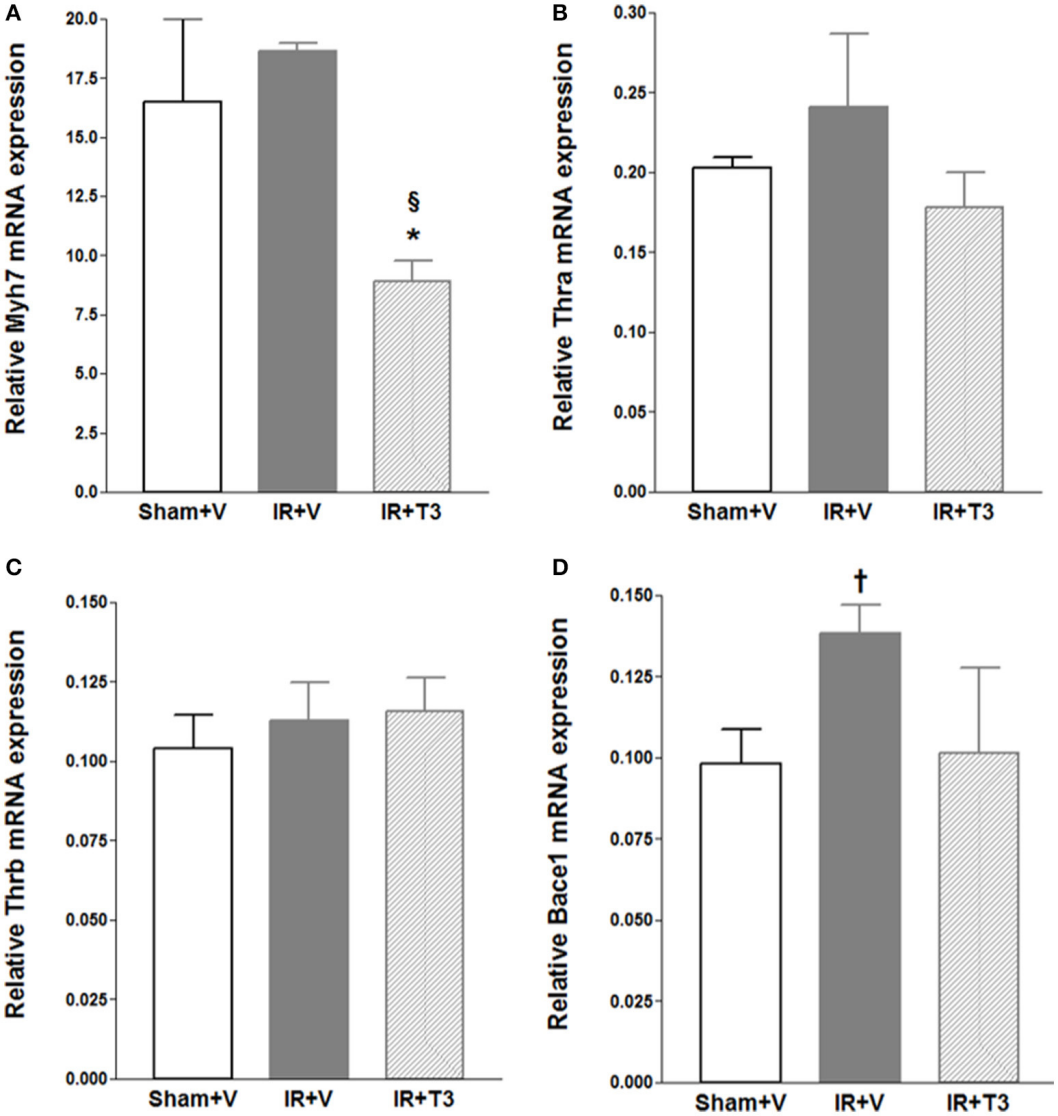
**Gene expression following oral (only) T3 treatment**. Values are means ± *SD*; V, Vehicle; IR, Ischemia-reperfusion; T3, Triiodo-L-thyronine. Gene expression was normalized using *cyclophilin A*. **(A)** Myh7, cardiac beta myosin heavy chains; **(B)** Thra, Thyroid hormone receptor alpha; **(C)** Thrb, Thyroid hormone receptor beta; **(D)** Bace1, β-site amyloid precursor protein cleaving enzyme 1; ^*^p < 0.05 vs. Sham+V (Anova, *post-hoc*); ^§^*p* < 0.05 vs. IR+V (Anova, *post-hoc*); ^†^*p* < 0.05 vs. Sham+V (*t*-test); *n* = 5 per group.

We investigated mRNA expression of representative genes relevant to IR and thyroid signaling (Figure 2). As expected, we found that beta myosin heavy chain (*Myh7*) expression was significantly downregulated following oral T3 treatment. Interestingly, β-site amyloid precursor protein cleaving enzyme 1 (*Bace1*) was increased following IR (*p* < 0.05) and returned close to sham levels following oral T3. MMP-2 and TIMP-1 levels did not significantly alter following T3 treatment (data not shown). TIMP-1 increased following T3 (Sham+V: 0.0306 ± 0.006; IR+V: 0.0342 ± 0.005; IR+T3: 0.067 ± 0.011, *p* < 0.05 vs. IR+V & Sham+V).

### Early initiation of continuous T3 treatment leads to safer and greater cardiac performance

**Figure 3:**
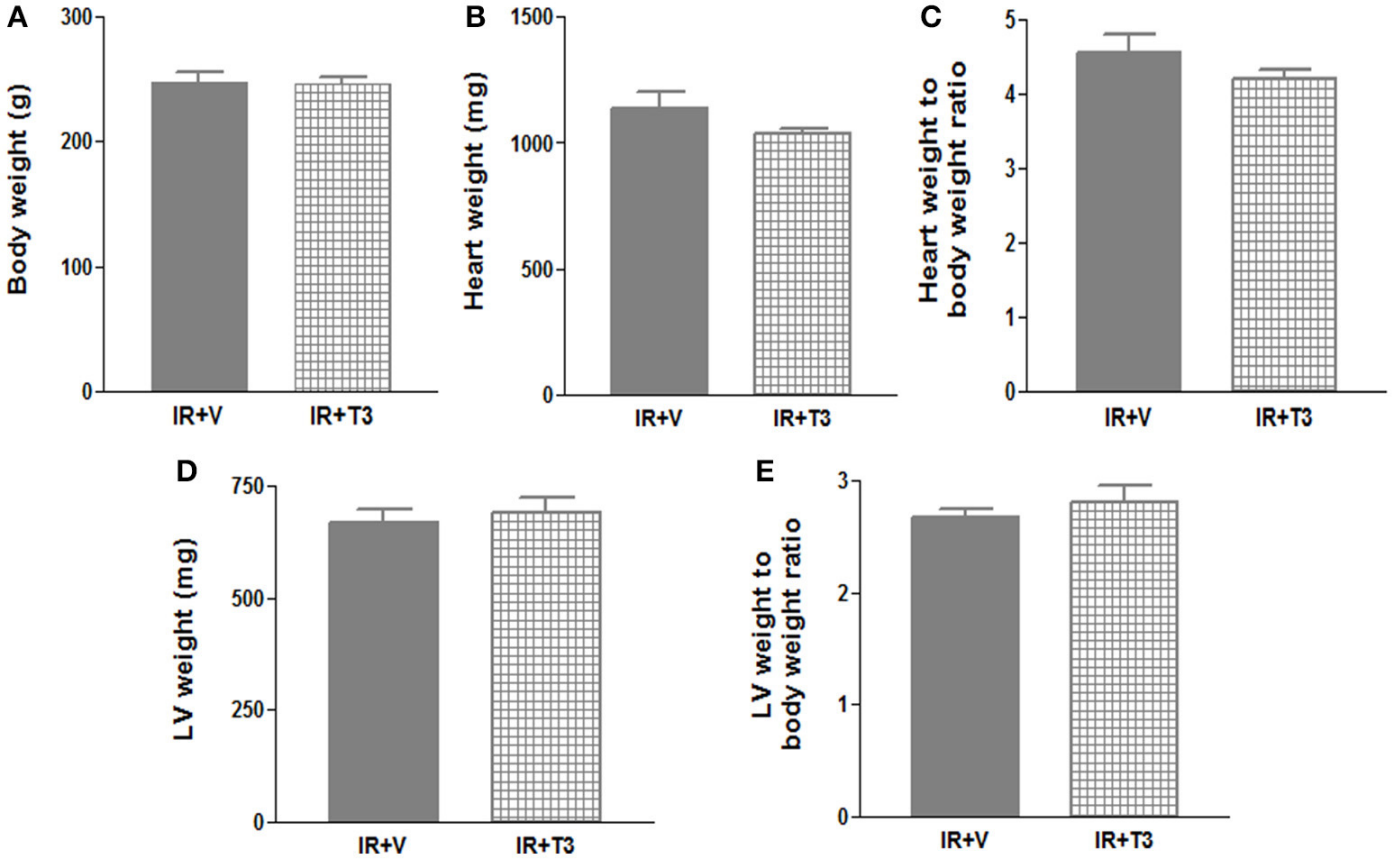
**No significant hypertrophy following continuous T3 (bolus + low-dose oral)**. **(A)** Body weight; **(B)** Heart weight; **(C)** Heart weight to body weight ratio; **(D)** LV weight; **(E)** LV weight to body weight ratio; Values are means ± *SD*; LV, Left ventricular plus septal; V, Vehicle; IR, Ischemia-reperfusion; T3, Triiodo-L-thyronine (12 μg/kg bolus followed by 4.5 μg/kg/day); *n* = 8–9 (IR+V), *n* = 7 (IR+T3).

**Figure 4:**
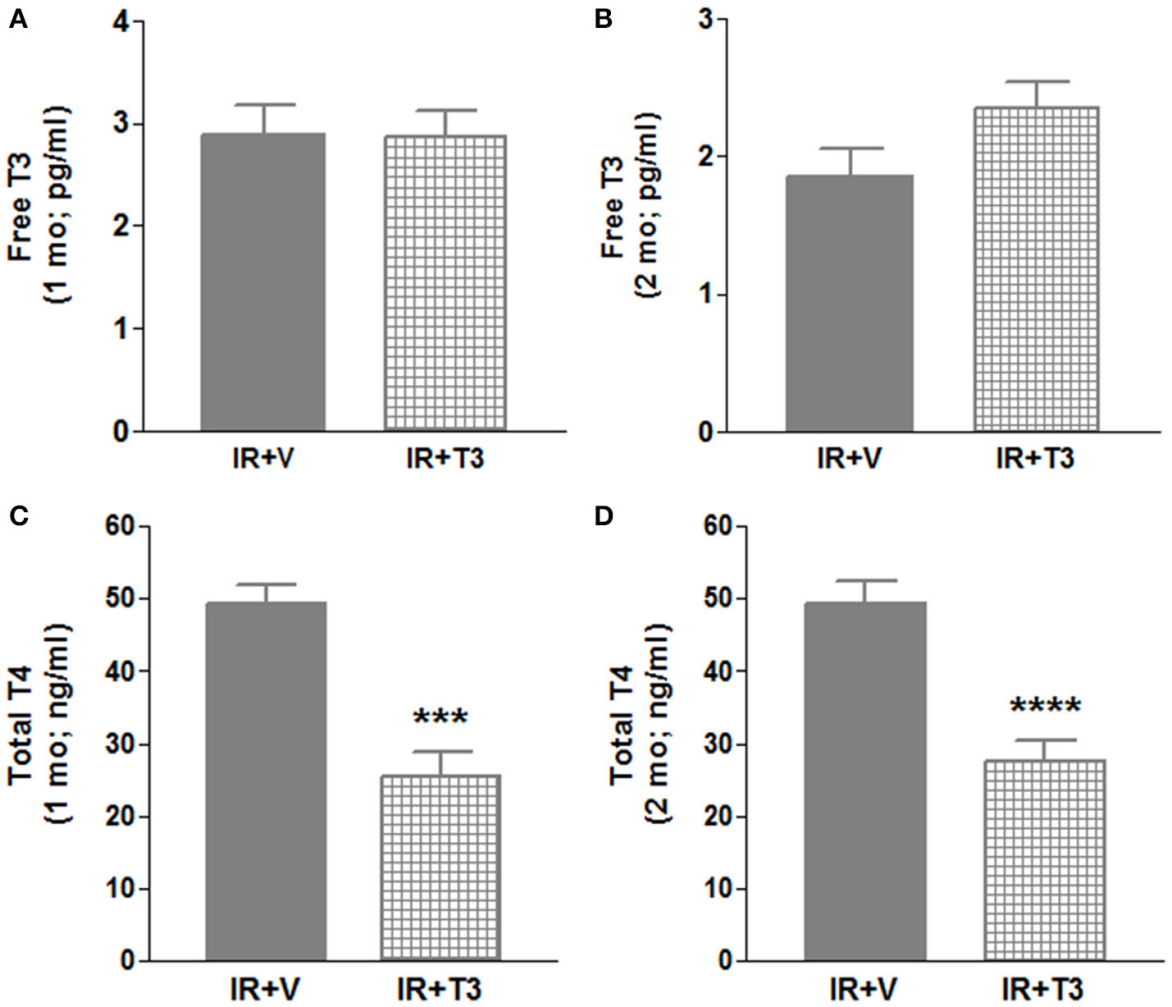
**Serum thyroid hormone levels in 2 mo IR rats after continuous T3 (bolus + low-dose oral)**. **(A)** Free T3 (1 mo); **(B)** Free T3 (2 mo); **(C)** Total T4 (1 mo); **(D)** Total T4 (2 mo); Values are means ± *SD*; V, Vehicle; IR, Ischemia-reperfusion; T3, Triiodo-L-thyronine; T4, Thyroxine; mo, month; ^***^p < 0.001, ^****^p < 0.0001; 1 mo: *n* = 5 (IR+V), *n* = 8–11 (IR+T3); 2 mo: *n* = 10–12 (IR+V), *n* = 11 (IR+T3).

Our observations have revealed that it takes about 6 h post-surgery for rats to start drinking water, and therefore, drug intake (Rajagopalan et al., 2016). To avoid this delay in availability of T3, we investigated whether administering a bolus T3 dose immediately following IR is safe and could result in enhanced cardiac performance. In a separate cohort of IR rats, we administered 12 μg/kg T3 i.p. upon reperfusion. Given that 8 μg/kg/d resulted in increased heart weight and free T3 levels (preceding experiment), we reduced the oral dose to 4.5 μg/kg/d, which was continued for 2 months following the i.p. treatment. We found that heart weights and LV weights were not significantly increased following this reduced T3 regimen (Figure 3) indicating no T3-induced hypertrophy. While serum total T4 levels showed feedback inhibition, the levels of serum free T3 were preserved (Figure 4).

**Figure 5:**
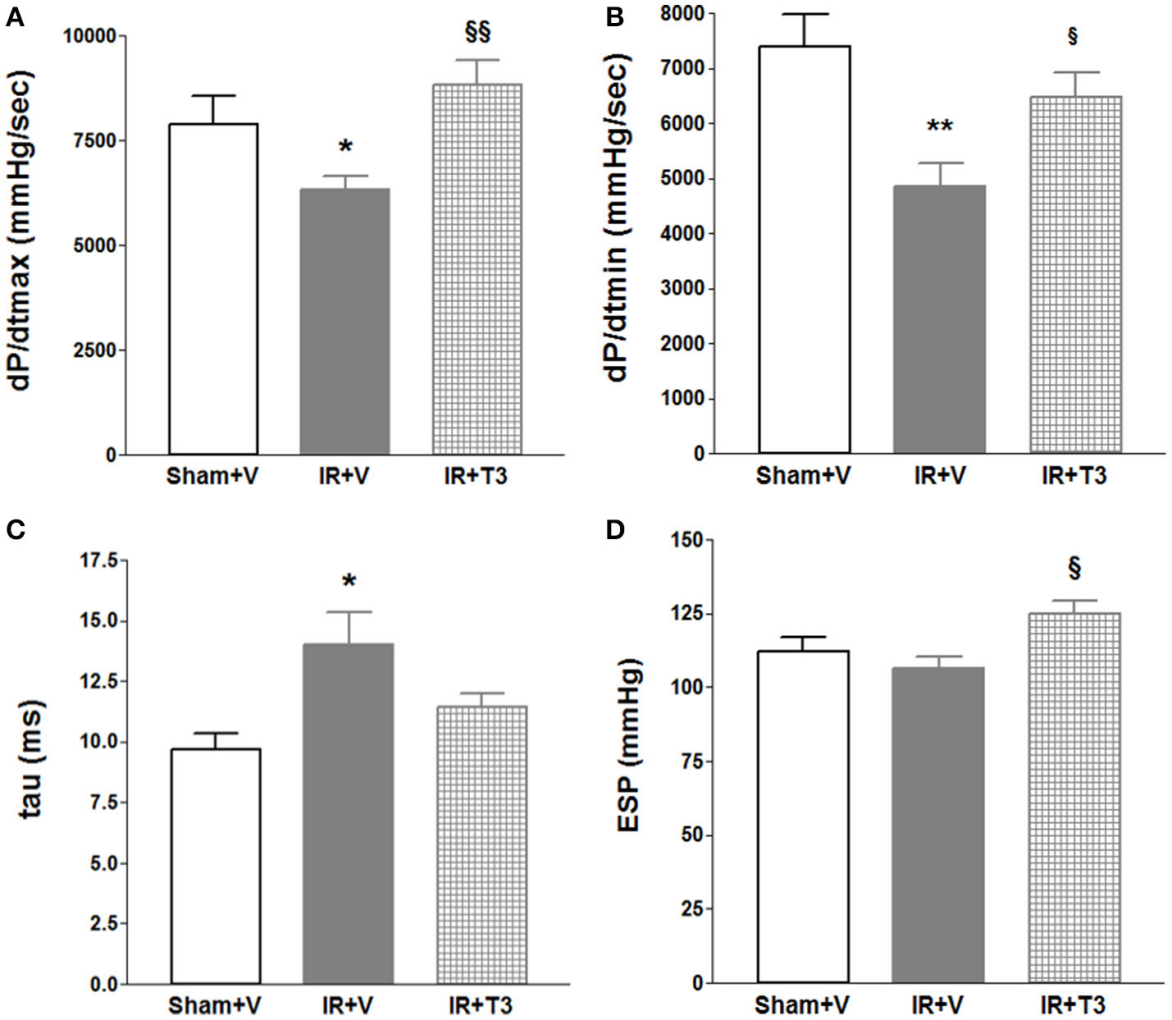
**Significant hemodynamic improvement after continuous (bolus + low-dose oral) T3 treatment**. Continuous T3 treatment following IR using combined peri-operative intraperitoneal and subsequent oral T3 treatment significantly improved **(A)** dP/dt_max_, maximal rate of pressure development and **(B)** dP/dt_min_, maximal rate of pressure decline. **(C)** tau, τ, LV relaxation time constant partially decreased and **(D)** ESP, LV End-systolic pressure increased; V, Vehicle; *n* = 7 (Sham+V), *n* = 11 in (IR+V); *n* = 9 in (IR+T3); ^*^p < 0.05, ^**^p < 0.01 vs. corresponding IR+V groups; ^§^*p* < 0.05, ^§§^*p* < 0.01 between both IR+T3 groups.

Compared to vehicle, continuous T3 treatment (bolus + low-dose oral) resulted in significant increases in dP/dt_max_, a measure of cardiac contractility (Figure 5; Supplementary Figure 2). Tau decreased by 19% following continuous T3 treatment (bolus + low-dose oral) and end-systolic pressures increased. Importantly, the increase in dP/dt_max_ was greater than that observed with oral T3 alone in the previous experiments in Figure 1A (*p* < 0.05) using a slightly higher dose. Compared to oral T3 (7.6 ± 1.5 mmHg), end-diastolic pressures following bolus + low-dose oral T3 (5.9 ± 0.84 mmHg) treatment were reduced, although not significant. There was no significant difference in HR between vehicle-treated and continuous T3-treated (bolus + low-dose oral) groups. Electrophysiological studies showed that two out of nine vehicle-treated IR rats developed significant inducible atrial arrhythmias (1.39 ± 1.3 s) compared to none in the continuous T3-treated group.

### Expression of long non-coding RNAs (lncRNAs)

Following IR, real-time qPCR-based lncRNA expression of H19 (0.0055 ± 0.004 vs. 0.0017 ± 0.001; *p* = 0.05) increased compared to sham. Continuous T3 treatment (0.0041 ± 0.002) did not significantly affect the expression levels compared to IR. There were no statistically significant differences in expression of other lncRNA targets including Tug1, Neat1, and Sra1 in this model (data not shown).

## Discussion

The new findings of the study include: a modified low-dose continuous oral T3 treatment-monitoring protocol significantly improved IR myocardial deficits without significant cardiac side effects. We also demonstrated a safe therapeutic window below the threshold of inducing increased heart mass or heart rate, information important in planning future clinical trials. Timely reperfusion of the infarct-related coronary artery is important for minimizing myocardial damage, infarct size, morbidity, and mortality (Bagai et al., 2014). These studies offer important new insight on key parameters including cardiac safety, efficacy, timing, and frequency of TH treatment. Together with our other studies in rat models of permanent coronary ligation (Rajagopalan et al., 2016), diabetes mellitus (Weltman et al., 2014), and hypertension (Weltman et al., 2015), we have now determined that ~3–6 μg/kg/day is a safe and efficacious treatment window for rats fed ad lib. Doses within this range offer significant improvements in cardiac contractility, relaxation, rhythm, and gene expression profile. Above this dose range, increases in HR and/or heart weight become detectible, suggesting a safe upper limit for desired treatment effects was reached. There was no evidence of any adverse health effects in the first IR study reported here using 8 μg/kg/day T3. Our findings also document for the first time, decreased atrial tachyarrhythmias in IR compared to MI (Rajagopalan et al., 2016). We speculate this may be due to lack of significant atrial dilation following IR compared to MI.

We determined whether T3 supplementation begun immediately upon reperfusion (IP bolus injection) provided further improvements over delayed oral T3 treatment alone (e.g., 6-h delay from commencement of drinking after surgery). This combination strategy showed greater improvement in cardiac contractility compared to oral treatment alone. This demonstrates that TH-mediated myocardial protection in the “early” reperfusion period is critical for preserving and further enhancing cardiac performance. Since blood pressures were not significantly altered (Supplementary Figure 3), these changes are likely the result of direct effects of T3 on the heart. These findings are supported by relevant studies in (i) large mammals of transient regional myocardial ischemia and reperfusion, (ii) transient global myocardial ischemia in patients undergoing cardiac surgery on cardiopulmonary bypass, and (iii) transient inadequate global myocardial perfusion in brain-dead potential organ donors (Novitzky and Cooper, 2014). Following administration of THs in these acute conditions, myocardial dysfunction associated with loss of high-energy phosphates and accumulation of tissue lactate was reversed. Although total T3 is frequently used, free T3, is now understood to be a clinically important biomarker in cardiovascular disorders and is the active form of the hormone (Iervasi et al., 2003; Fontana et al., 2012; Cerillo et al., 2014; Kishi, 2015; Jankauskiene et al., 2016; Wang et al., 2016). While 8 μg/kg/day showed increased serum free T3 levels, 4.5 μg/kg/day did not, indicating that the lower dose is both a safer cardiac dose and free T3 may be a clinically relevant biomarker for monitoring during T3 therapy. Although there was no further increase in salvaged myocardium despite increased Timp1 (Kinoshita et al., 2014) compared to IR, T3 significantly enhanced cardiac functional performance along with reduced beta myosin heavy chain levels. Other studies have shown reduced infarct characteristics with THs (Forini et al., 2014; Rajagopalan et al., 2016). However, compared to the present study, they differ with nature of ischemia, duration of infarction, duration of treatment, etc.

Numerous studies from our group and others have extensively documented TH-mediated cardioprotective mechanisms. We demonstrated that T3/T4 protects the heart and vascular system via several mechanisms, viz., sarcomeric, thyroid, ion channels, fibrosis, sympathetic, mitochondria, autophagy, growth factors, cell structure, etc. (Liu and Gerdes, 1990; Tang et al., 2005; Chen et al., 2012, 2013; Zhang et al., 2013; Weltman et al., 2014; Rajagopalan et al., 2016). Myocardial contraction results from coordinated cross-bridging of sarcomeric myosin heavy chains and these are regulated by T3 at the promoter level (Kahaly and Dillmann, 2005). In the present study, as demonstrated before (Chen et al., 2013; Weltman et al., 2013, 2014), we found that beta myosin heavy chain expression was significantly decreased by oral T3. This indicates that the T3-mediated improvement in cardiac function is at least partially mediated at the sarcomeric level. T3 is also a negative regulator of the most abundant cardiac TH receptor, *Thra* (Kahaly and Dillmann, 2005; Pantos and Mourouzis, 2014), and our data showed a downward trend in expression levels with oral T3. Ischemia contributes to hypoperfusion and inflammation. One of the markers following brain ischemia is increased *Bace1*, β-site amyloid precursor protein cleaving enzyme 1 (Nural-Guvener et al., 2013; Sun et al., 2015). Although *Bace1* inhibition attenuates neuroinflammation (Neumann et al., 2015), its role in myocardial ischemia and TH-mediated cardioprotection is unknown. We found that *Bace1* levels are increased in IR LV and oral T3 returned them closer to control levels. This indicates that *Bace1* may be a potential target and worthy of future investigation. We have previously shown that T3 protects cardiomyocytes against ischemia-induced apoptosis via Akt signaling (Chen et al., 2008). Others have also demonstrated role of oxidative stress in subclinical hypothyroidism (Obradovic et al., 2016). Using *in vivo* and isolated cardiomyocyte approaches, Forini et al. (2014) recently showed that subcutaneous infusion of T3 restored non-coding microRNA miR-30a expression, downregulated p53 and Bax, limited mitochondrial membrane depolarization and decreased apoptosis and necrosis in the IR area at risk. Along with short non-coding microRNAs, interest in the role of long non-coding RNAs in cardiovascular health and disease has increased. As was shown in mouse models of hypertrophic HF (Lee et al., 2011) and human HF (Greco et al., 2016), we found that rat H19 lncRNA levels were increased following IR injury.

### Limitations

As discussed elsewhere (Zhang et al., 2014), female rats were used because they maintain a more stable body mass over time than males, thus minimizing interpretation of heart weight alterations related to this potential distractor. Females also have a higher incidence of thyroid anomalies requiring TH replacement (Sara et al., 2015). Since other studies have demonstrated TH-based cardioprotective effects in males (Pantos et al., 2008; Henderson et al., 2009), a different outcome would be unexpected. T3 treatment in a sham group was not included because producing hyperthyroidism has been studied extensively and was not a goal here (Gerdes et al., 1987; Venditti et al., 2002; Kuzman et al., 2005; Weltman et al., 2012; Zhang et al., 2013). After more than 30 years of examining myocyte remodeling using the most accurate available methods (e.g., Coulter Channelyzer analysis of isolated myocytes and direct measurement of isolated myocyte length), we have found that LV mass changes exceeding ~15% are generally needed to reach statistical significance in myocyte remodeling parameters (Gerdes and Pingitore, 2014). Since current data show values less than this limit, we did not assess myocyte remodeling. Furthermore, as the goals of the study centered on the heart, we did not study non-cardiac effects. Long-term survival studies would be of future interest and we anticipate beneficial effects with T3 treatment under serum monitoring. Based on our experience from several hundred consistent coronary ligation microsurgeries, we obtained only representative transverse mid wall LV and not multiple LV sections (Chen et al., 2008, 2013; Rajagopalan et al., 2016). This is consistent with observations of others (Kanamori et al., 2011; Eulalio et al., 2012).

## Conclusions

The study provides a modified, treatment-monitoring protocol for T3 treatment of myocardial IR injury in rats. This information should provide helpful guidance for further clinical studies. We optimized both the dose and timing and have shown that lower doses of oral T3 delivered from the onset of IR injury provide the greatest benefits with no obvious cardiac side effects. T3 is commercially available and inexpensive. The translational nature of this study provides encouraging supportive data for ongoing (http://ponte-project.eu/) and future clinical trials.

## Author contributions

All experiments were conducted in the laboratory of AG at the New York Institute of Technology College of Osteopathic Medicine, Old Westbury, NY, USA. Authors AG, VR, CP, and OS provided conception and design of the work. Authors VR, YZ, CP, CC, SS, AL, OS, and YC contributed to acquisition, analysis or interpretation of data. VR wrote the manuscript.

## Funding

Research reported in this publication was supported by the National Heart, Lung, and Blood Institute of the NIH, Bethesda, MD, under Award Number R01HL103671 (AG). The content is solely the responsibility of the authors and does not necessarily represent the official views of the National Institutes of Health.

### Conflict of interest statement

The authors declare that the research was conducted in the absence of any commercial or financial relationships that could be construed as a potential conflict of interest.
