# Supplementary material for: Modified Low-Dose Triiodo-L-thyronine Therapy Safely Improves Function Following Myocardial Ischemia-Reperfusion Injury
Source: Front Physiol. 2017 Apr 12;8:225. doi: 10.3389/fphys.2017.00225 (PMC5388763; doi:10.3389/fphys.2017.00225)
Supplement: Supplementary file 1 [file DataSheet1.docx]

***Supplementary Material***

**Modified Low-dose Triiodo-L-thyronine Therapy Safely Improves Function Following Myocardial Ischemia-Reperfusion Injury**

Viswanathan Rajagopalan, PhD^1*^, Youhua Zhang^2^, MD, PhD, Christine Pol^2^, PhD, Clifford Costello^2^, BSE, Samantha Seitter^2^, BS, Ann Lehto^2^ BS, Olga V Savinova^2^, PhD, Yue-feng Chen^2^ MD, PhD, A Martin Gerdes^2^, PhD

*** Correspondence:** Corresponding Author: [vrajagop@nyit.edu](mailto:vrajagop@nyit.edu)

**Supplementary figure 1: Cardiac histology**


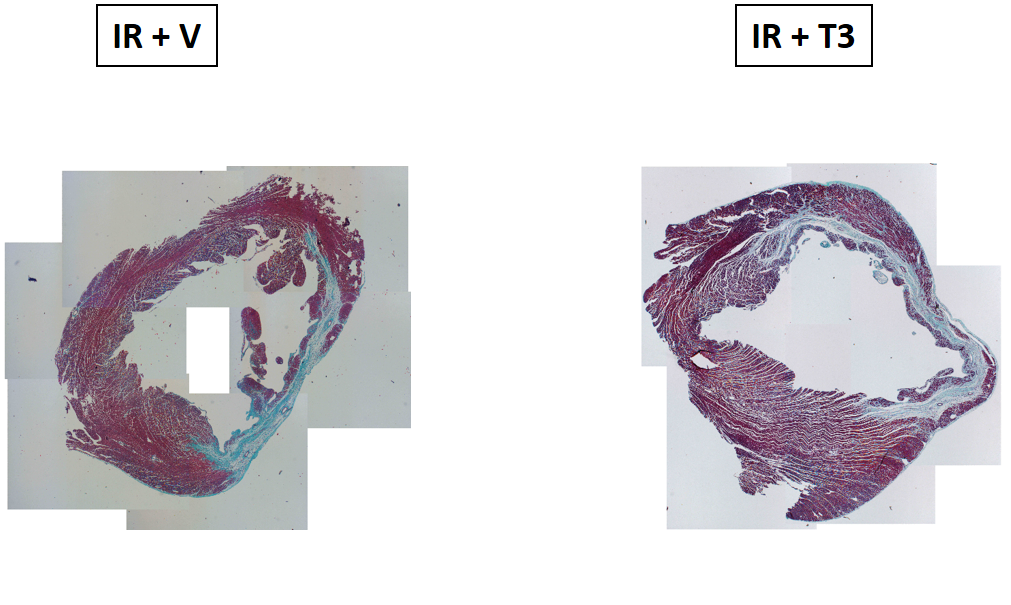


Representative histological sections (transverse mid-left ventricular 2x) following trichrome staining showing infarct characteristics with oral T3 treatment. V – Vehicle; IR – Ischemia-reperfusion; T3 – Triiodo-L-thyronine

**Supplementary figure 2: Pressure tracings**


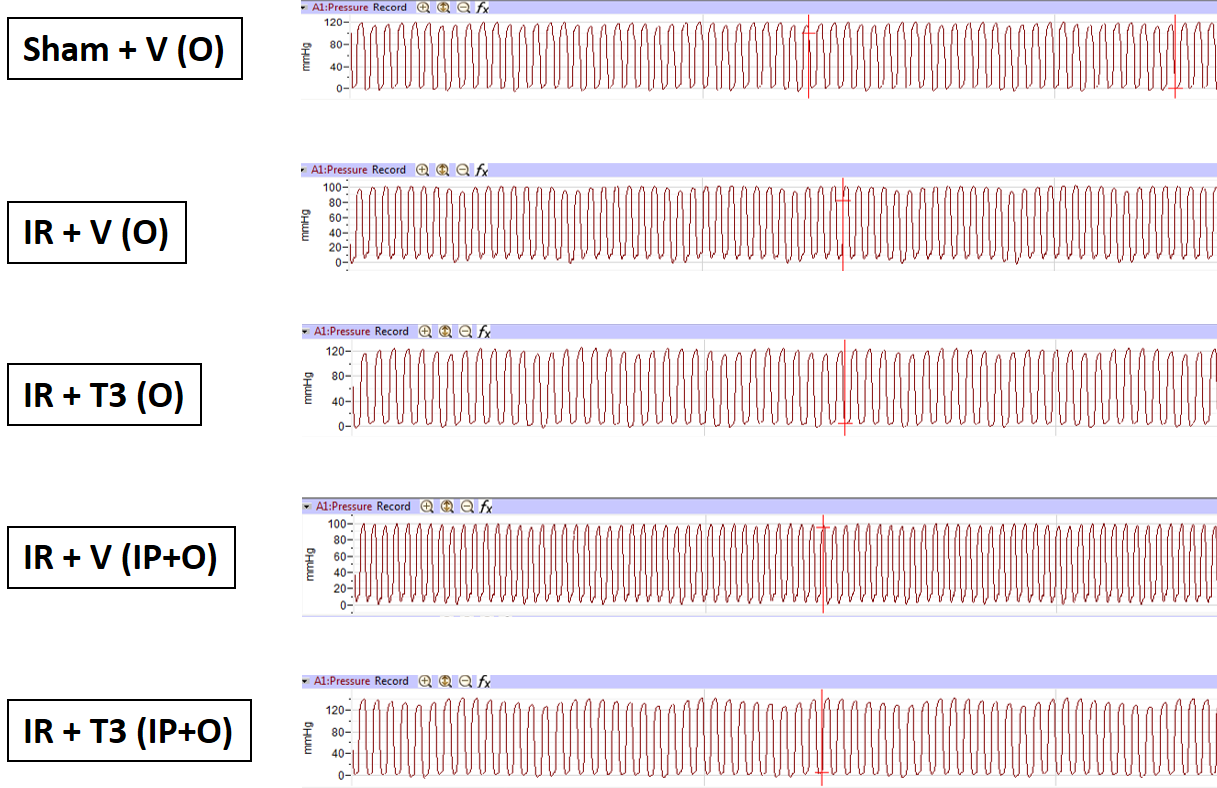


Representative left ventricular pressure tracings with different T3 treatment protocols. V – Vehicle; IR – Ischemia-reperfusion; T3 – Triiodo-L-thyronine; IP – Intraperitoneal; O – Oral

**Supplementary figure 3: No significant change in blood pressures following T3 treatment.**


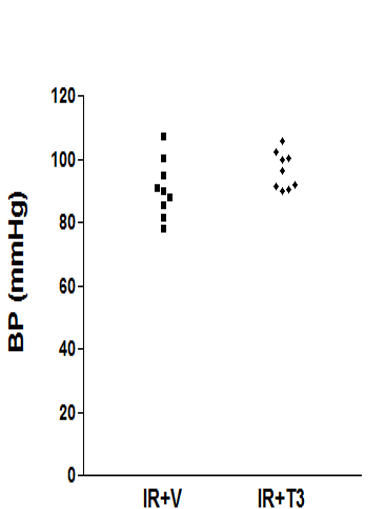


Mean arterial pressures in vehicle and T3-treated (intraperitoneal and oral) rats subjected to IR. V – Vehicle; IR – Ischemia-reperfusion; T3 – Triiodo-L-thyronine

**Supplementary table:**

| **Description** | **Gene Name** | **Symbol** | **Accession** |
| --- | --- | --- | --- |
| Glyceraldehyde-3-phosphate dehydrogenase | BARS-38, Gapd | gapdh | NM_017008 |
| Beta actin | Actx | actb | NM_031144 |
| Peptidylprolyl isomerase A (cyclophilin A) | CYCA, CyP-A | ppia | NM_017101 |
| Thyroid hormone receptor alpha | ERBA1, thra1, c-erbA-1 | thra | NM_031134 |
| Thyroid hormone receptor beta | C-erba-beta, ERBA2, Nr1a2, RATT3REC, T3rec, TRbeta | thrb | NM_012672 |
| Myosin, heavy chain 7, cardiac muscle, beta | Bmyo, Myhcb, myHC-beta, myHC-slow | myh7 | NM_017240 |
| Beta-site APP cleaving enzyme 1 | Bace | bace1 | NM_019204 |
| Matrix metallopeptidase 2 | - | mmp2 | NM_031054 |
| TIMP metallopeptidase inhibitor 2 | - | timp2 | NM_021989 |
| TIMP metallopeptidase inhibitor 1 | TIMP-1, Timp | timp1 | NM_053819 |
| H19, imprinted maternally expressed transcript (non-protein coding) | ASM, ASM1, D11S813E | h19 | NR_027324 |
| Taurine-upregulated 1 lncRNA | - | Tug1 | KT943446 |
| Steroid receptor RNA activator 1 |  | Sra1 | NM_183329 |
| Nuclear paraspeckle assembly transcript 1 (non-protein coding) | - | neat1 | AC_000069 |

Accession numbers of targets used for expression studies.
